# Diagnosis and Dental Management of a Patient With Forbes Albright Syndrome: A Report of a Rare Case

**DOI:** 10.7759/cureus.62208

**Published:** 2024-06-11

**Authors:** Harshita Lath, Gaurav Patri, Atul Bajoria, Arun K Mohanty, Debkant Jena

**Affiliations:** 1 Department of Conservative Dentistry and Endodontics, Kalinga Institute of Dental Sciences, KIIT (Deemed to be University), Bhubaneswar, IND; 2 Department of Oral Medicine and Radiology, Kalinga Institute of Dental Sciences, KIIT (Deemed to be University), Bhubaneswar, IND; 3 Prosthodontics, Kalinga Institute of Dental Sciences, KIIT (Deemed to be University), Bhubaneswar, IND; 4 Department of Conservative Dentistry and Endodontics and Endodontics, Kalinga Institute of Dental Sciences, KIIT (Deemed to be University), Bhubaneswar, IND

**Keywords:** xerostomia, hyperprolactinemia syndrome, forbes-albright syndrome, dental management, cabergoline

## Abstract

Forbes Albright syndrome is a hyperprolactinemia syndrome linked to a pituitary tumor associated with galactorrhea and amenorrhea. Cabergoline, an ergot derivative, is its drug of choice.

Here, we report the oral manifestations and management of a case of a 32-year-old female, diagnosed and treated with the same. The patient had an alarming increase in the incidence and progression of dental caries. Her diagnosis and management have been highlighted.

This can have overbearing effects on the psychology and function of the individual, thus making early diagnosis and precise management important.

## Introduction

Forbes Albright syndrome is an uncommon hyperprolactinemia syndrome. It is a rare condition, occurring in less than 1% of the general population [1], linked to a pituitary tumor associated with galactorrhea and amenorrhea. It is characterized by increased prolactin secretion, enlarged pituitary gland, galactorrhea, absence of menstruation, and lack of ovulation [2].

The primary therapy for patients with hyperprolactinemia and prolactinomas is treatment with a dopamine agonist such as bromocriptine or cabergoline. These drugs normalize prolactin levels and significantly reduce the tumor volume in most patients, and extensive experience has shown their usefulness in treating prolactinomas of all sizes [3].

Oral manifestations are early and easy detectors of a disorder. They pave the pathway for timely, noninvasive diagnosis compared to any other organ system. The most common disorder afflicted on the tooth is dental caries. It is also the primary reason for a patient to visit a dental clinic. Dental caries stems from an imbalance between tooth demineralization and remineralization. Xerostomia is an important causative factor for the same, among others.

In this article, we report the dental management of a patient whose diagnosis of the syndrome was established after taking a detailed medical and drug history and collaborating this history with the etiology of the sudden onset of rampant caries. An opinion from a medical expert (endocrinologist) was also taken.

The utilization of pharmaceutical agents to manage the syndrome may exacerbate oral manifestations, thus making it imperative for interdisciplinary collaboration between dental practitioners and healthcare providers to optimize therapeutic outcomes with minimal adverse effects.

## Case presentation

An Asian female patient, aged 32 years, reported to the Department of Conservative Dentistry and Endodontics, with a complaint of rampant decay of teeth over six months. It was associated with mild pain, difficulty in chewing, and esthetic concerns.

On further investigation of past medical, the patient was revealed to be having an irregular menstrual cycle and was diagnosed with hyperprolactinemia, galactorrhea, and pituitary tumor one year back. Drug history presented the prescription of cabergoline for a year.

**Figure 1:**
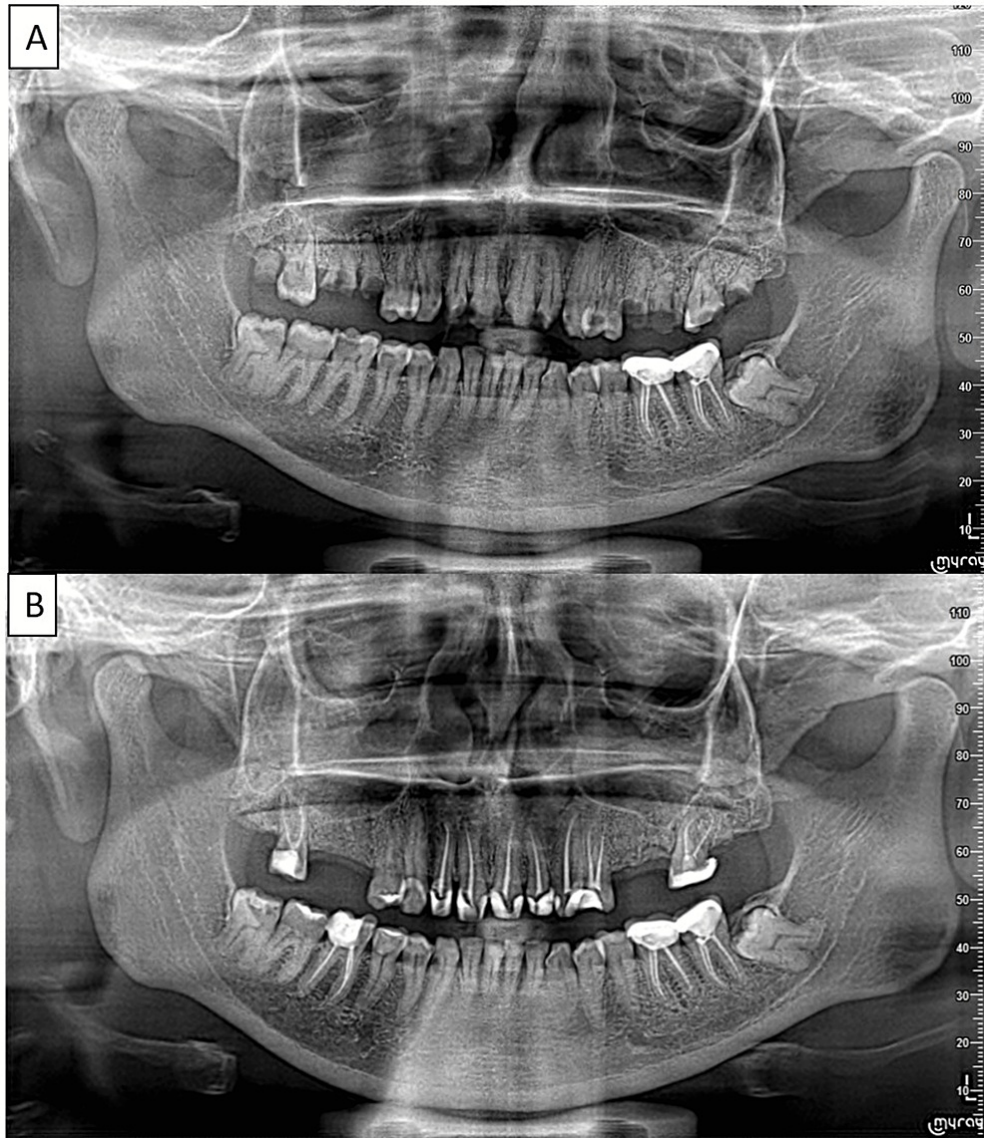
(A) Preoperative OPG; (B) post-RCT OPG. OPG, orthopantomogram; RCT, root canal treatment

Hard tissue examination revealed deep dentinal caries with respect to (wrt) 11, 12, 17, 21-24, 27, 46; moderate dentinal caries wrt 14, 47; root stumps wrt 15, 16, 18, 25, 26, 28; and prosthesis wrt 36 and 37. A vitality test with an electric pulp tester (Waldent, India) showed that tooth 14 was vital, while teeth 11, 12, 17, 21-24, 27, and 46 were nonvital. An orthopantomogram (OPG) was advised (Figure 1A).

It is noteworthy that the incidence of caries was noticed over a brief period of six months. To ascertain a reason for it, quantitative and qualitative salivary examinations were done. After locating the salivary duct orifice, the salivary flow was assessed with pre-weighed Wharton’s filter paper. It was noted that the salivary flow was reduced. The consistency of the saliva was thick and ropy.

After a thorough evaluation of her medical history and required investigations, a meeting was set up with an endocrinologist where we came up with the diagnosis of Forbes Albright syndrome.

A diagnosis of irreversible pulpitis with symptomatic apical periodontitis was made for 11, 12, 15-8, 21-28, and 46 with xerostomia. There was no evidence of any super-added oral infection.

Treatment planning was done in phases. There was no requirement for emergency treatment. Patient education, oral prophylaxis, and the extraction of teeth 15, 16, 18, 25, 26, and 28, followed by root canal treatment (RCT) of teeth 11, 12, 17, 21-24, 27, and 46 were planned in phase 1. Restoration of teeth 14 and 47 followed by prosthetic rehabilitation was scheduled for phase 3. A maintenance phase compromising quarterly checks for 12 months was proposed. The treatment plan was discussed with the patient and valid consent was obtained.

Phase 1 treatment was initiated with plaque control, patient education, and oral prophylaxis. After extraction of the aforementioned teeth and adequate healing, RCT was initiated for teeth 11, 12, 21, 22, 23, and 24. After adequate anesthesia with 2% lidocaine hydrochloride, caries were removed under rubber dam isolation, exposing the pulp chamber. Canal instrumentation and shaping were performed up to size F4 with ProTaper Gold files (Dentsply Maillefer, Ballaigues, Switzerland), irrigated with 5.25% sodium hypochlorite. Laser-assisted sterilization was conducted, followed by cold lateral compaction obturation using AH Plus sealer (Dentsply-Sirona, York, PA, USA). Temporary restoration (Cavit, 3M ESPE, Seefeld, Germany) was applied.

The patient was evaluated for any post-op symptoms one week later. In the subsequent sitting, composite restorations using Solar Sculpt (GC, Tokyo, Japan) were performed for teeth 11, 12, 21, 22, 23, and 24. Teeth 14 and 47 were restored with giomer (Beautifil II, Coltene, Germany). For 46, RCT and composite build-up were done.

For 17 and 27, caries excavation led to more than 50% of tooth structure loss, necessitating a post and core build-up after RCT. RCT was performed following the same procedure as previously described. Sectional obturation was done in the palatal canal and a fiber post (Coltene, Germany) was placed. The core build-up was done with fiber-reinforced composite cubes (Fibrafill, Dentapreg, Tallinn, Estonia), a de-coupling matrix (Dentapreg UFM), and nanohybrid composite (Solar Sculpt, GC). A postoperative OPG was done after endodontic intervention (Figure 1B).

**Figure 2:**
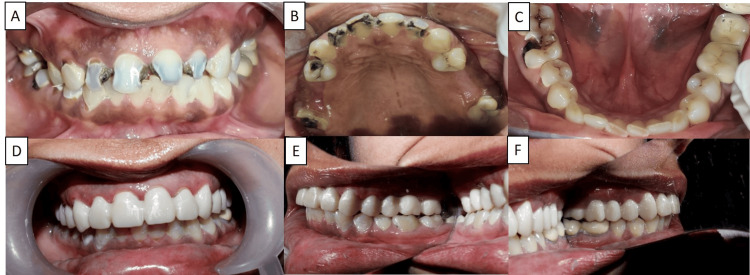
(A-C) Preoperative intraoral pictures; (D-F) postoperative intraoral pictures.

A full-mouth rehabilitation complete with face bow transfer and ceramic crowns followed the endodontic and restorative intervention. Pretreatment and posttreatment clinical pictures are shown in Figures 2A-2F.

To manage xerostomia, vitamin C supplements and artificial saliva mouth rinse were prescribed.

The patient is currently under quarterly follow-ups for a year.

## Discussion

To the best of our knowledge, this is the first case report that describes the dental manifestations and related management of a patient with Forbes Albright syndrome. Dopamine therapy is used in prolactin-secreting macro-adenomas since it is one of the primary regulators of pituitary prolactin production. It works through dopamine D2 receptors to suppress prolactin production [2]. The primary therapy for such patients is bromocriptine or cabergoline, with the latter showing more successful results [3].

Apart from being the drug of choice for this syndrome, cabergoline is also used in managing Parkinson’s disease. The Canadian Dental Association has listed cabergoline as a drug causing xerostomia [4]. In a multicenter study examining the efficacy and tolerability of long-term cabergoline therapy in hyperprolactinemic disorders, Webster et al. [5] encountered dry mouth as a symptom in a few patients.

The potential decrease in saliva observed with cabergoline use may be attributed to the drug's action on dopamine receptors, which can affect the autonomic nervous system and glandular secretions. However, the specific impact on saliva production can vary among individuals [6].

We propose that due to low salivary secretion, the patient had an increased incidence and progression of dental caries. This is a result of a decrease in the pH of saliva and the proliferation of cariogenic bacteria, namely Streptococcus mutans and Lactobacillus species. The decay is most often recurrent or primary and located at sites generally not usually susceptible to caries [7]. Thus, it was important to select appropriate restorative materials with caries preventive action. There is no evidence of the association of cabergoline with dental caries in scientific literature.

Giomers are fluoride-releasing, light-cured restorative materials that represent a true hybrid of composite and glass-ionomer cement (GIC). They have surface pre-reacted glass (S-PRG) technology. They have demonstrated their ability to prevent caries through fluoride release (from the glass-ionomer component) while maintaining the mechanical and esthetic properties of composite resins [8]. Hence, they were used as the restorative material of choice for moderate carious lesions in this case.

A Fibrafill cube is a resin composite with an integrated continuous membrane that prevents stress concentration and reduces the risk of crack development and propagation. According to the manufacturer, for teeth with less remaining structure, Fibrafill increases the fracture toughness of the build-up.

It is proven that laser-assisted root canal irrigation has superior efficacy in the elimination of microorganisms, dentin debris, and smear layers from the root canal system [9].

Oral manifestations of this syndrome were managed by a multidisciplinary healthcare team that helped the patient maintain oral hygiene and dental health.

Since the drugs used in syndromes cannot be replaced, their adverse effects need to be diagnosed, recorded, and treated to prevent further damage. Patient counseling and education to improve cognitive realization and self-awareness are paramount.

## Conclusions

This case addressed the oral clinical manifestations of Forbes Albright syndrome and difficulties experienced during diagnosis and dental management. It suggests the importance of correlating the symptoms to the medical history and emphasizing the underlying etiology rather than simply addressing the present objective and subjective findings. Knowledge of the loop-mediated association among oral complications of this syndrome is essential to perfecting treatments.
